# In Operando GISAXS and GIWAXS Stability Study of Organic Solar Cells Based on PffBT4T‐2OD:PC_71_BM with and without Solvent Additive

**DOI:** 10.1002/advs.202001117

**Published:** 2020-07-01

**Authors:** Dan Yang, Franziska C. Löhrer, Volker Körstgens, Armin Schreiber, Bing Cao, Sigrid Bernstorff, Peter Müller‐Buschbaum

**Affiliations:** ^1^ Lehrstuhl für Funktionelle Materialien Physik‐Department Technische Universität München James‐Franck‐Str. 1 Garching 85748 Germany; ^2^ Department of Chemistry University of Alberta Edmonton AB T6G 2G2 Canada; ^3^ Elettra—Sincrotrone Trieste S.C.p.A. Strada Statale 14‐km 163.5 in AREA Science Park, Basovizza Trieste 34149 Italy; ^4^ Heinz Maier‐Leibnitz Zentrum (MLZ) Technische Universität München Lichtenbergstr. 1 Garching 85748 Germany

**Keywords:** crystallinity, degradation, in operando, organic photovoltaics, solvent additives

## Abstract

Solvent additives are known to modify the morphology of bulk heterojunction active layers to achieve high efficiency organic solar cells. However, the knowledge about the influence of solvent additives on the morphology degradation is limited. Hence, in operando grazing‐incidence small and wide angle X‐ray scattering (GISAXS and GIWAXS) measurements are applied on a series of PffBT4T‐2OD:PC_71_BM‐based solar cells prepared without and with solvent additives. The solar cells fabricated without a solvent additive, with 1,8‐diiodoctane (DIO), and with *o*‐chlorobenzaldehyde (CBA) additive show differences in the device degradation and changes in the morphology and crystallinity of the active layers. The mesoscale morphology changes are correlated with the decay of the short‐circuit current *J*_sc_ and the evolution of crystalline grain sizes is codependent with the decay of open‐circuit voltage *V*_oc_. Without additive, the loss in *J*_sc_ dominates the degradation, whereas with solvent additive (DIO and CBA) the loss in *V*_oc_ rules the degradation. CBA addition increases the overall device stability as compared to DIO or absence of additive.

## Graphical abstract

The influence of solvent additives on operation‐induced changes of the morphology in the active layer of organic solar cells is studied in operando. High boiling point solvent additives introduce an interpenetrating network of donor and acceptor in the films and change the device degradation mechanism from a short‐circuit current dominated one to a open‐circuit voltage dominated degradation.

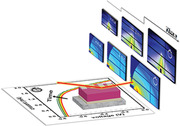

Solar energy harvested with organic solar cells (OSCs) is considered as a promising potential alternative renewable and green energy sources due to the low costs, flexibility, solution processing, and large‐scale fabrication.^[^ ^1^, ^2^ ^]^ Since many years, increasing the power conversion efficiency (PCE) of champion devices is in the main research direction of organic photovoltaics (OPVs).^[^ ^3^, ^4^ ^]^ Now the champion PCE for single‐junction OSCs is around 16%,^[^ ^5^, ^6^, ^7^, ^8^ ^]^ which is quite close to the value of theoretical predictions.^[^ ^9^ ^]^ However, despite high PCE values reached so far, a poor device stability is seriously blocking the real application of OSCs today.^[^ ^10^, ^11^ ^]^ As a consequence, a better understanding of the degradation mechanism of OSCs is attracting more and more attention.^[^ ^12^, ^13^, ^14^ ^]^ In general, the various degradation processes occurring in OSCs can be roughly divided into two branches, namely chemical and physical degradation processes.^[^ ^10^, ^15^ ^]^ The chemical degradation is mainly attributed to the reaction between water and oxygen with the materials in the device.^[^ ^16^, ^17^ ^]^ Hence, encapsulation techniques and more stable materials are being developed to avoid these problems. In contrast, physical degradation is considered to arise mainly from the morphology deterioration of bulk heterojunction (BHJ) films.^[^ ^10^ ^]^ So far, two major morphological degradation pathways have been determined: a demixing‐driven coarsening of inner structures and a mixing‐driven loss of connectivity due to shrinkage of domains.^[^ ^18^, ^19^ ^]^ Recent experiments indicated more complexity in the morphological degradation^[^ ^20^ ^]^ and thus the operation‐driven morphology changes in BHJ devices still need to be further explored.

Solvent additives are widely used to modify the morphology of BHJ films to achieve a good interpenetrating network of donor/acceptor (D/A) materials for an improved exciton dissociation and charge transport.^[^ ^21^, ^22^, ^23^, ^24^, ^25^, ^26^ ^]^ A solvent additive usually has two features, namely that the boiling point is higher than that of the host solvents, and one component of the donor and acceptor blend is selectively dissolved in the solvent additive.^[^ ^27^, ^28^ ^]^ It has been reported in our previous work that the residual solvent additive in the final device leads to an obvious decay of the fill factor (FF) during the operation when the leftover solvent is escaping from the device under vacuum conditions.^[^ ^18^, ^20^ ^]^ Therefore, avoiding the presence of residual solvent in the final devices should be taken into consideration to avoid such degradation pathway.

In earlier work, PTB7‐Th:PC_71_BM films were fabricated at room temperature and no post treatments were involved in the device fabrication process.^[^ ^20^ ^]^ In the present work, poly[(5,6‐difluoro‐2,1,3‐benzothiadiazol‐4,7‐diyl)‐alt‐(3,3′′′‐di(2‐octyldodecyl)‐2,2′,5′,2′′,5′′,2′′′‐quaterthiophen‐5,5′′′‐diyl)] (PffBT4T‐2OD) and [6,6]‐phenyl‐C71‐butyric acid methyl ester (PC_71_BM) are chosen as the donor and acceptor materials, respectively. The PffBT4T‐2OD:PC_71_BM films are spin‐coated at 110 °C, followed by thermal annealing at 85 °C for 5 min under nitrogen atmosphere. The annealing step is selected to remove the solvent additives to a large extent. Moreover, PffBT4T‐2OD is a highly crystalline polymer,^[^ ^29^, ^30^, ^31^ ^]^ which is beneficial for a high charge carrier mobility.^[^ ^32^, ^33^, ^34^ ^]^ We fabricate solar cells without solvent additive, with 1,8‐diiodoctane (DIO, boiling point at 332 °C) and with *o*‐chlorobenzaldehyde (CBA, boiling point at 212 °C), respectively. From the comparison of the degradation behavior, we learn about the impact of the type of solvent additive.

In operando grazing‐incidence small/wide angle X‐ray scattering (GISAXS/GIWAXS) characterizations are successfully used to determine the correlation between the evolution of BHJ morphology and the photovoltaic device performance.^[^ ^18^, ^19^, ^35^, ^36^, ^37^ ^]^ In the present study, in operando GISAXS and GIWAXS are sequentially performed on a series of PffBT4T‐2OD:PC_71_BM‐based devices, fabricated without and with solvent additives, to reveal the operation‐induced changes of the nanostructures and crystallinity in the BHJ layers during the aging process. The measurement chamber, the experimental protocol, and other experimental details are described in the Supporting Information. Notably, all devices are made without any special encapsulation and the experiment chamber is evacuated to avoid degradation caused by the presence of oxygen and moisture in the air during the measurements.

The temporal evolution of the normalized photovoltaic parameters of the devices detected during the in situ GIWAXS measurements is displayed in **Figure** **1**. Since the device performance has been tracked during the in situ GISAXS and in situ GIWAXS measurements individually, two sets of temporal evolution of the photovoltaic parameters are obtained. Figure S1, Supporting Information, shows the respective temporal evolution of the normalized photovoltaic parameters of the devices recorded during the in situ GISAXS experiments. Small individual differences in the decrease of the parameters are seen in particular in the initial burn‐in phase. At later stages of the device degradation, the overall temporal characteristics agree well for the in operando GISAXS and GIWAXS experiments. The individual differences arise from the well‐known spread in the photovoltaic parameters among different devices of the same type. The average photovoltaic performance of all devices is shown in Table S1, Supporting Information, as measured immediately after fabrication in ambient in the laboratory. The PffBT4T‐2OD:PC_71_BM solar cells fabricated with DIO have higher PCE values than the other devices. Doping with CBA cannot further enhance the device performance, which is attributed to the small difference of the boiling points between 1,2‐dichlorobenzene (DCB, 180 °C) and CBA (212 °C). However, comparing the temporal evolution of the normalized PCE values of all devices (Figure 1), it can be seen that the devices with CBA are the most stable and the devices with DIO show the strongest decay of the device performance. To make the comparison clearer, we model the PCE decay curves via exponential functions (Figure S2, Supporting Information) and find that 85% of the PCE maintain even after 2000 min in the device with CBA treatment, but only 66% of the PCE preserves in the device with DIO additive, which directly indicates that a solvent additive can affect the device stability. The 10% loss of the short circuit current (*J*_sc_) is the main degradation factor in the device fabricated without a solvent additive.

**Figure 1:**
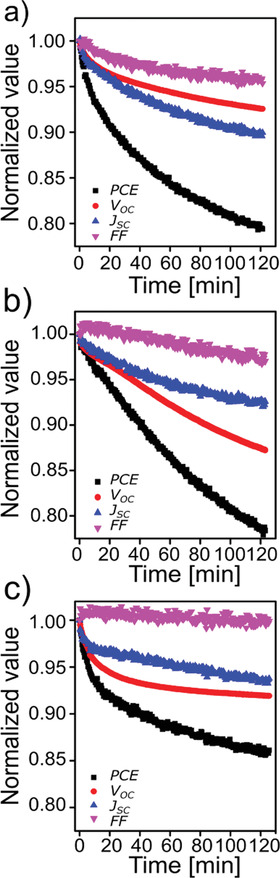
Temporal evolution of PCE (black), FF (purple), *J*_SC_ (blue), and *V*_OC_ (red) of the devices a) without solvent additive, b) with DIO additive, and c) with CBA additive, respectively.

An efficient charge carrier separation and transport highly depends on the sizes of the nano‐structured domains in the BHJ layer.^[^ ^38^, ^39^ ^]^ As the exciton diffusion length is typically in the range of 10–20 nm for conjugated polymers, the domain sizes determine if excitons can reach the D/A interface to dissociate. Thus, the inner nanostructure of the active layer directly impacts on the device performance.^[^ ^19^, ^40^ ^]^

Changes of the morphology of the BHJ layer are tracked on a nanometer scale during the device degradation process via in operando GISAXS. Figure S3, Supporting Information, displays the 2D GISAXS data of each device at selected times in the operation process. Detailed observations of morphological changes are determined via extracting the scattering signals of PffBT4T‐2OD at the Yoneda^[^ ^41^ ^]^ region via horizontal line cuts of the 2D GISAXS data. The horizontal line cuts are shown in Figure S4, Supporting Information. To evaluate the nanostructures of the polymer, these horizontal line cuts are modeled based on the effective interface approximation and the local monodisperse approximation (details are given in the Supporting Information).^[^ ^42^, ^43^ ^]^ In our modeling (red curves in Figure S4, Supporting Information), three characteristic cylinder nanostructures are applied for the samples with and without solvent additive. The average radii of the polymer domains at the respective times are plotted for each device in **Figure** **2**. It can be seen that the initial radii of the largest polymer domains in the sample without solvent additive is around 11.5 ± 0.3 nm, which is smaller than that in the devices with DIO (15.5 ± 0.3 nm) and CBA (15.0 ± 0.4 nm) additives. The slight increase in the polymer domain sizes in the devices with solvent additives is due to polymer aggregation in solution. Thus, the addition of DIO and CBA leads to a morphology modification in the active layers, which agrees well with previous reports.^[^ ^21^, ^44^, ^45^ ^]^ During operation, the average radii of the largest polymer domains increase with time in the device fabricated without solvent additive (Figure 2a). Contrary no obvious changes of the polymer domain radii are visible in the devices with DIO (Figure 2b) and CBA (Figure 2c) additives. It has been claimed that solvent additives, having a high boiling point and selectively dissolving PC_71_BM, provide a better integration of the PC_71_BM molecules into the donor polymer aggregates.^[^ ^44^, ^46^ ^]^ The more pronounced interpenetration between donor and acceptor molecules in the system with an additive gives less opportunity for the migration of molecules, which can explain the more stable BHJ morphology for devices with DIO and CBA additive. We conclude that the continuous decay of *J_sc_* in the device without an additive is induced by the increase of polymer domains with time, probed in the GISAXS measurements, which leads to a decrease of the chances for charge carrier separation. The differences in the decay of *J*_sc_ between the devices with DIO and CBA solvent additive illustrate the complexity of the morphology impact on individual device parameters. Besides the simple size of the polymer domains also other morphology parameters such as their connectivity and crystallinity are of importance.

**Figure 2:**
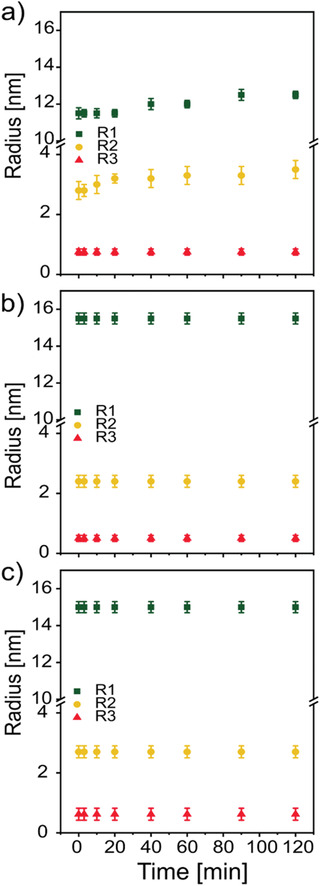
Temporal evolution of the average radii of three characteristic polymer structures in the device a) without solvent additive, b) with DIO additive, and c) with CBA additive.

In earlier work, the loss of residual solvent additives from the BHJ layer was identified to cause a shrinkage of the polymer domains, which reduced the connectivity of domains and caused a decrease of the FF.^[^ ^18^, ^20^ ^]^ In contrast in the present work, the FF is observed to be the most stable photovoltaic parameter in the devices fabricated with solvent additive (DIO or CBA, Figure 1b,c), which we attribute to having almost no more additive left inside the active layers to be removed during the device operation. Actually, we detect no solvent additive losses from the vertical line cuts of the 2D GISAXS data (Figure S5, Supporting Information), because the distance and amplitude of the resonant diffuse scattering along the *q_z_* direction are almost stable during the entire measurement time. In contrast, the decrease of the open circuit voltage (*V*_oc_) dominates the device degradation in the devices with solvent additive (DIO or CBA), as shown in Figure 1. *V*_oc_ is impacted by many factors, such as recombination rates, density‐of‐state (DOS) shape, charge carrier, and exciton mobility in organic photovoltaics.^[^ ^47^, ^48^ ^]^ All these impact factors are closely related with the crystalline state of the materials in the BHJ layer.^[^ ^35^, ^49^, ^50^ ^]^ Thus, in operando GIWAXS measurements are applied to reveal changes of the crystalline parts of the active layers during the in operando measurements.

Figure S6, Supporting Information, shows the collected 2D GIWAXS data of each device at selected times during operation. Cake cuts of the 2D GIWAXS data are performed to extract the respective crystalline information (see Supporting Information). The pronounced (100) Bragg peak located at *q_z_* = 0.27 Å^−1^ is ascribed to the PffBT4T‐2OD crystallites, whereas the polymer (010) Bragg peak (1.72 Å^−1^) and the fullerene peak (1.30 Å^−1^) are both weak in intensity and broad (Figure S7, Supporting Information). Thus, an edge‐on orientation of the polymer crystallites is dominant in these films while the face‐on orientation is strongly suppressed. Changes in crystallinity caused by device operation are seen from a comparison of the initial and the final vertical cake cuts of the device with DIO as example, since the polymer (010) Bragg peak is only observed for this device (Figure S8, Supporting Information). We find that the PC_71_BM peak and the polymer (010) Bragg peak are almost constant during the measurements, whereas the polymer (100) Bragg peak changes. Consequently, we determine changes of the polymer crystalline state during the operation process by tracking the PffBT4T‐2OD (100) Bragg peak. By fitting with Gaussian functions, we obtain the q‐position, the crystalline grain size (estimated via Scherrer equation) and the peak intensity for all devices (a fitting example is shown in Figure S9, Supporting Information, for the device made with CBA). The respective results are summarized in **Figure** **3**. It should be noted that the initial stage, marked in Figure 3 with grey dashed lines, is considered as a “burn‐in” phase, since the peak intensity shows a fast increase in the beginning of the operation for all samples.^[^ ^51^ ^]^ A “burn‐in” stage was reported in the literature before, while its origin is still debated. Oxygen trapped within the films, the broad polydispersity of the polymers used in the active layers, and organic or inorganic impurities in the polymers (such as palladium catalysts) were suggested as possible origin of the “burn‐in” phenomena.^[^ ^52^, ^53^, ^54^, ^55^, ^56^ ^]^ Since the “burn‐in” is followed by the device degradation, we will restrict to the aging in the present work. In Figure 3, it can be observed that the q‐position of the (100) Bragg peak stays almost unchanged during the operation process in all devices after the initial “burn‐in” stage. Thus, the polymer crystallites undergo no changes of the crystal lattice during operation after having passed the burn‐in.

**Figure 3:**
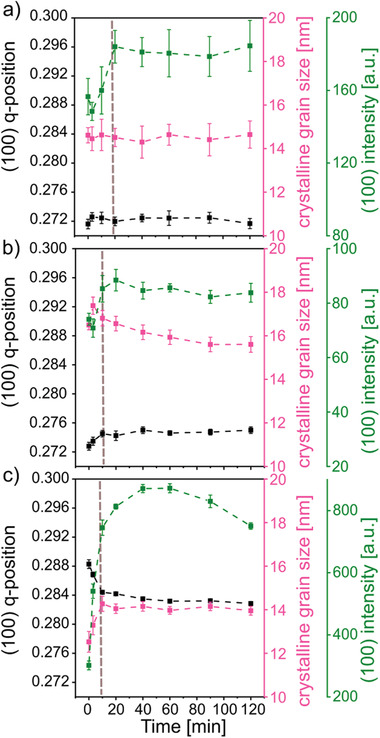
Temporal evolution of the PffBT4T‐2OD (100) Bragg peak in terms of peak intensity (green), peak position (black), and peak FWHM (pink) for devices a) without solvent additive, b) with DIO additive, and c) with CBA additive, respectively. The error bars are obtained from the fitting model. The dashed lines are as guides to the eye.

The crystalline grain sizes of PffBT4T‐2OD remain nearly constant in the devices without an additive and with CBA additive, whereas they decrease in the device with DIO additive. To further determine the influence of the applied bias voltage and the used vacuum conditions on the crystallinity of the BHJ film, a dark in operando measurement is carried out on another fresh DIO device. The bias voltage is periodically applied on the device without illumination and an in‐situ GIWAXS measurement is performed like for the illuminated device. The results of the dark in operando measurements are shown in Figure S10, Supporting Information. The crystalline grain sizes almost stay constant within the error bars after the burn‐in stage in the dark control experiment, which indicates that the morphological changes observed in the illuminated sample require the presence of voltage and illumination. It was reported in a previous study that a codependence exists between crystalline states of the BHJ layer and the *V*_oc_ value probed during the aging process.^[^ ^35^ ^]^ In **Figure** **4**, the crystalline grain sizes and *V*_oc_ values are plotted together for all three devices. We can see that the changes of *V*_OC_ show a reasonable correlation with the evolution of the crystalline grain sizes after the burn‐in stage. The crystal grain sizes and *V*_oc_ values in the device without an additive and with CBA additive are almost stable after the burn‐in stage, while the crystalline grain sizes and *V*_oc_ value show a correlated decrease in the device with DIO additive. Thus, the shrinkage of the crystalline grain sizes causes the observed decrease of the *V*_oc_ values in the device with DIO additive, as seen before in case of P3HT based devices.^[^ ^35^ ^]^ Moreover, the initial *V*_oc_ value of the device with DIO additive is 0.72 V, which is lower than that of the devices without an additive and with CBA additive (0.76 V, as shown in Table S1, Supporting Information). Based on the GIWAXS observations, we know that the crystallite sizes in the device with DIO additive are slightly larger than those present in the samples with CBA additive and without any additive. It was demonstrated that the donor crystallites with extended *π*‐conjugation could cause a slight increase of the highest occupied molecular orbital (HOMO) level of the donors and therefore result in a decrease of the *V*_oc_ value.^[^ ^57^, ^58^ ^]^ Furthermore, the lower degree of polymer crystallinity in the device with DIO additive, obtained by comparing the initial Bragg peak intensities of all devices (Figure 3), might lead to a lower *V*_OC_ value as well.^[^ ^59^ ^]^ In detail, we can also observe that the Bragg peak intensities almost remain constant in the devices without an additive and with DIO additive during operation. However, a slight decrease of Bragg peak intensity is recorded in the device with CBA additive, suggesting a decrease of the polymer crystallinity during the operation time and resulting in a loss of *J*_sc_. Because high crystallinity facilitates charge transport, the mobility of charge carriers in polymer crystallites is higher than that in amorphous domains.^[^ ^60^, ^61^ ^]^

**Figure 4:**
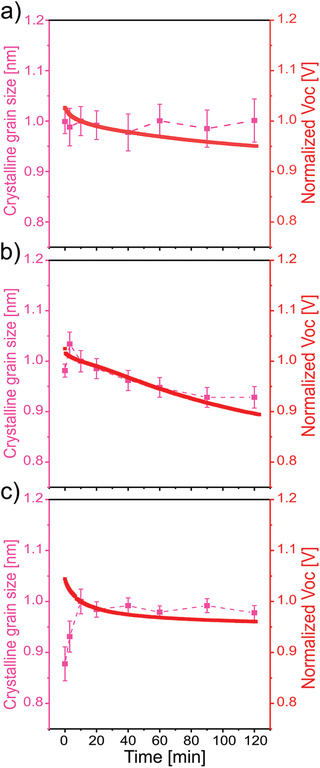
Comparison of the temporal evolution of the crystalline grain size (pink) in (100) direction versus *V*_OC_ (red) for devices a) without solvent additive, b) with DIO additive, and c) with CBA additive, respectively. The data are normalized to the value at 10 min. The dashed lines are guides to the eye.

The absolute peak intensities of the device with CBA additive are significantly higher than those of the devices without additive and with DIO additive (Figure 3). We propose that a competition of polymer molecules between forming interpenetrating D/A networks and growing crystallites is triggered by an additive in the BHJ films. Combining the results of GISAXS and GIWAXS measurements, we know that the polymer morphology experiences a phase separation (shown in **Figure** **5**a) during the device aging in the device without solvent additives, which causes a motion of polymer molecules.^[^ ^62^ ^]^ Therefore, the polymer domains become larger during the in operando measurements, resulting in the decay of the *J*_SC_. However, from the GIWAXS measurements we observe that the polymer crystallites are more stable in this device, which means that the growth of the domains is not caused by growing polymer crystals but by the addition of amorphous polymers. In contrast, from the use of a high boiling point additive like DIO, an interpenetrating D/A network is promoted (shown in Figure 5b), resulting in a better *J*_sc_ and FF (Table S1, Supporting Information).^[^ ^44^, ^63^ ^]^ A low boiling point solvent additive like CBA is beneficial for polymer molecule aggregation as well, but more favorable for polymer crystallite growth (shown in Figure 5c).^[^ ^46^ ^]^ Moreover, the extended film drying time in case of doping with solvent additives is also beneficial for forming face‐on orientated crystallites.^[^ ^64^ ^]^ The out‐of‐plane (010) and (100) Bragg peaks correspond to the face‐on and edge‐on crystallites of the polymer phase.^[^ ^65^ ^]^ Figure S7, Supporting Information, shows the initial vertical cake cuts of all devices. There are almost no face‐on crystallites in the BHJ film prepared without solvent additives, whereas, for the devices fabricated with solvent additives, the (010) Bragg peak becomes observable, especially in the sample with DIO additive.

**Figure 5:**
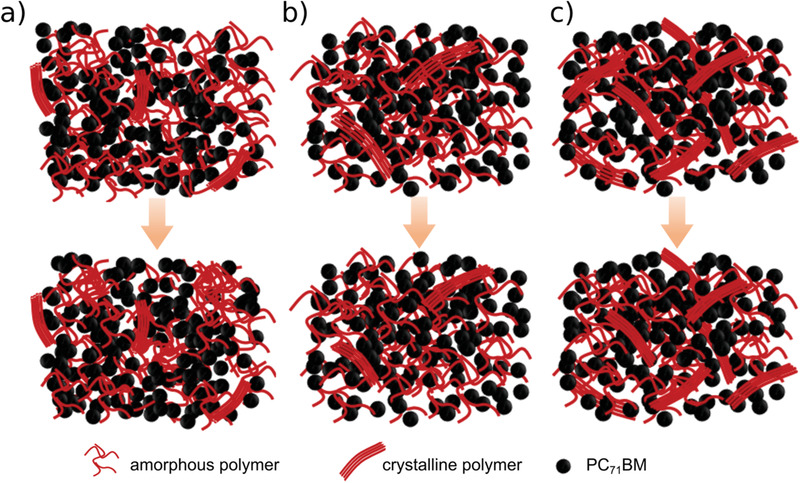
Schematic illustration of the operation‐induced changes of the morphology of PffBT4T‐2OD:PC_71_BM solar cells a) without solvent additive, b) with DIO additive, and c) with CBA additive. The initial morphology (top row) changes after 120 min of operation (bottom row) as described in the text.

In summary, we investigate the impact of solvent additives on the stability of PffBT4T‐2OD:PC_71_BM solar cells with in operando GISAXS and GIWAXS measurements. Due to post‐production annealing we ensure that no substantial residual solvents are released during the device operation. In case of PffBT4T‐2OD:PC_71_BM solar cells, doping with solvent additives helps to obtain more donor and acceptor interpenetrating networks in the BHJ layers to form a stable morphology, which is reflected by a stable FF. However, solvent additives also can cause competition between forming a D/A interpenetrating network and crystallite growth in the donor polymer molecules. DIO enhances the polymer's ability to form interpenetrating networks in the BHJ film, but it lowers the polymer crystallinity, and results in a lower *V*_oc_. Thus, the DIO additive turns out to be most beneficial for the performance of PffBT4T‐2OD:PC_71_BM solar cells. However, the best stability is present in devices with CBA additive, because these devices have a higher polymer crystallinity in the active layer. Thereby, our work gives a further understanding of the degradation mechanism in high‐efficiency OSCs based on solvent additives. The findings can help for choosing a suitable solvent additive, which can well balance the interpenetrating network character and the crystallinity in a BHJ film for next generation solar cells.

## Experimental Section

##### Materials

Poly[(5,6‐difluoro‐2,1,3‐benzothiadiazol‐4,7‐diyl)‐alt‐(3,3′′′‐di(2‐octyldodecyl)‐2,2′,5′,2′′,5′′,2′′′‐quaterthiophen‐5,5′′′‐diyl)] (PffBT4T‐2OD) was purchased from California organic semiconductors Inc., the molecular weight (*M*_w_) was around 131 kDa, and [6,6]‐phenyl‐C71‐butyric acid methyl ester (PC_71_BM) was purchased from 1‐materials Inc. All solvents, which were used in this work, including the solvent additives DIO and CBA were obtained from Sigma‐Aldrich and all chemicals were used as received without any further purification.

##### Device Fabrication

For device fabrication, indium‐doped tin oxide (ITO) coated glass substrates (8–12 Ω per sq) were sequentially cleaned via ultra‐sonication in Alconox, ethanol, acetone, and isopropyl alcohol, each for 10 min. Afterward, the substrates were dried with N_2_ and treated in an O_2_‐plasma cleaning step (Plasma‐System‐Nano, Diener Electronic GmbH). The ZnO precursor solution, which was prepared following an earlier report,^[^ ^66^ ^]^ was spin‐cast at 3000 rpm on top of the cleaned ITO substrates, and subsequently annealed at 150 °C for 30 min in air, resulting in a transparent ZnO thin film with a thickness of around 30 nm. A mixture of PffBT4T‐2OD: PC_71_BM (1:1.2, 20 mg mL^−1^ in total) was dissolved in a combination of 1,2‐dichlorobenzene (DCB) and chlorobenzene (CB) (1:1, 3% DIO or 5% CBA) while stirring and heating (100 °C) overnight. Prior to spin‐coating a BHJ layer, the ITO substrates with ZnO film were heated for 1 min at 120 °C, then the hot PffBT4T‐2OD: PC_71_BM solution was dropped onto the ZnO layer, the time interval between placing a substrate and starting spin‐coating was around 10 s. Afterward, the samples were annealed under nitrogen atmosphere at 85 °C for 5 min. Finally, a thin layer of MoO_3_ (about 10 nm) and a layer of Al (60 nm) were deposited successively via thermal evaporation under vacuum conditions. The active area of each pixel of the devices was about 12 mm^2^.

##### Film Characterization

In operando GISAXS and GIWAXS measurements were performed at the Austrian SAXS beamline of the Elettra Sincrotrone Trieste, at an X‐ray energy of 8 keV. For the GISAXS measurements, the incidence angle was set to 0.35° and the sample‐detector distance to 1365.5 mm. The DPDAK software^[^ ^67^ ^]^ was applied to extract the polymer scattering signals. For the GIWAXS measurements, the incidence angle was around 0.3° with a sample‐detector distance changed to 296 mm. The obtained 2D scattering patterns were evaluated using the GIXSGUI software.^[^ ^68^ ^]^

## Conflict of Interest

The authors declare no conflict of interest.

## Supporting information

Supporting Information
