# Supplementary material for: In Operando GISAXS and GIWAXS Stability Study of Organic Solar Cells Based on PffBT4T‐2OD:PC71BM with and without Solvent Additive
Source: Adv Sci (Weinh). 2020 Jul 1;7(16):2001117. doi: 10.1002/advs.202001117 (PMC7435237; doi:10.1002/advs.202001117)
Supplement: Supplementary file 1 — Supporting Information [file ADVS-7-2001117-s001.pdf]

Supporting Information

In-operando GISAXS and GIWAXS stability study of organic solar cells based on PffBT4T-2OD:PC71BM with and without solvent additive

*Dan Yang, Franziska C. Löhrer, Volker Körstgens, Armin Schreiber, Bing Cao, Sigrid Bernstorff, Peter Müller-Buschbaum**

D. Yang, F. C. Löhrer, Dr. V. Körstgens, A. Schreiber, Prof. P. Müller-Buschbaum
Lehrstuhl für Funktionelle Materialien, Physik-Department, Technische Universität München.
James-Franck-Str. 1, 85748 Garching, Germany
E-mail: muellerb@ph.tum.de

Dr. B. Cao
University of Alberta, Department of Chemistry, Edmonton, Canada

Dr. S. Bernstorff
Elettra – Sincrotrone Trieste S.C.p.A., Strada Statale 14 – km 163.5 in AREA Science Park, Basovizza, 34149 Trieste, Italy

Prof. P. Müller-Buschbaum
Heinz Maier-Leibnitz-Zentrum, Lichtenbergstr. 1, 85748 Garching, Germany

Keywords: in-operando, organic photovoltaic, degradation, solvent additive, crystallinity

Performance of solar cells

Table S1. The average photovoltaic performances of PffBT4T-2OD: $PC_{71}BM$ devices without and with solvent additives.

	J_{SC} (mA/ cm^2)	*Voc* (V)	*PCE* (%)	*FF*
without	9.3±0.3	0.76±0.01	3.4±0.1	0.48±0.01
DIO	18.1±0.2	0.72±0.01	7.7±0.1	0.58±0.01
CBA	10.0±0.1	0.76±0.01	3.8±0.2	0.48±0.01

*average values are taken from 8 or more devices.

In-operando chamber and GISAXS/GIWAXS measurement

The used measurement setup was introduced in our previous work in detail.[1] To collect the morphology evolution, grazing incidence small and wide angle X-ray scattering (GISAXS / GIWAXS) and current-voltage measurements were simultaneously performed on the devices. The chamber was maintained at vacuum conditions (p ~ 6×10^{-2} mbar) during the operation process to avoid degradation via oxygen and water. A PerkinElmer PX5 150 W xenon arc lamp was used to simulate a sun radiation spectrum illuminating the solar cells, and the photovoltaic performance was recorded by a source meter (Keithley 2400). Two Kapton windows on the front and backside of the chamber allowed the X-ray beam to enter and exit the chamber. To avoid the strong scattering signals from the metal electrode, the beam was aligned to probe the active layer film close to the electrode (1 mm distance from metal contact).

Before the in-operando experiments, two-dimensional (2D) GISAXS / GIWAXS data were measured to characterize the initial device state (denoted as 0 min). Afterwards, the devices were illuminated, and current-voltage curves were recorded periodically every 26 s for 120 minutes in total. In parallel, 2D GISAXS / GIWAXS data were taken after 3, 10, 20, 40, 60, 90 and 120 min of illumination with an exposure time of 5 s each.

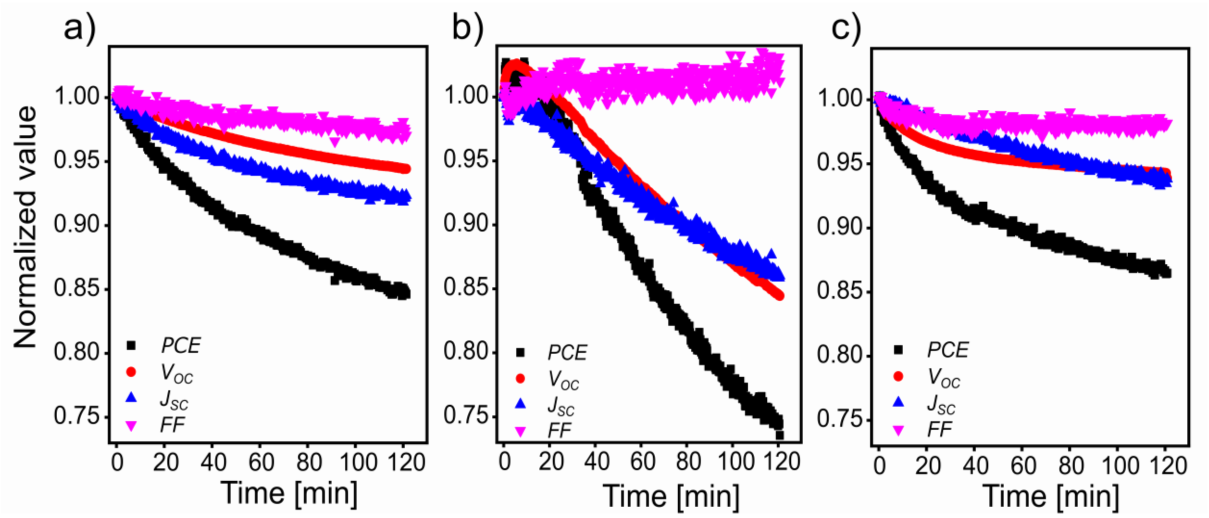

Figure S1. Temporal evolution of *PCE* (black), *FF* (purple), J_{SC} (blue) and V_{OC} (red) of the devices without solvent additive (a), with DIO additive (b) and with CBA additive (c) during in-operando GISAXS measurements, respectively.

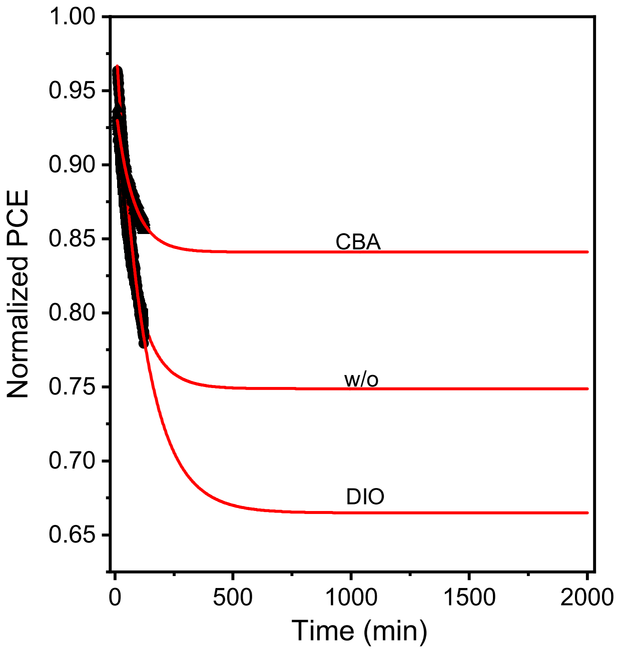

Figure S2. Extending the temporal evolution of the PCE decay curves (black symbols) from the measurements towards times t >> 120 min with modelling via exponential functions (red lines). The corresponding devices are indicated.

GISAXS measurements and analysis

Figure S1 shows the 2D GISAXS images of device without additive (Figure S1a), with DIO additive (Figure S1b) and with CBA additive (Figure S1c) at selected times during the in-operando process. To extract the scattering signal of the polymer PffBT4T-2OD, horizontal line cuts were performed at the Yoneda peak position of PffBT4T-2OD.

The effective interface approximation of the distorted wave Born approximation (DWBA), which works well for modeling GISAXS data of polymer films, is chosen to model the scattering data in the small angle regime. In addition, to deal with data containing several average sizes of objects, the local monodisperse approximation (LMA) is applied,[2,3] which is based on the assumption that in local domains with the size of the coherence length of the beam, only monodisperse objects are present. In this model, the total scattering signal can be approximated by the incoherent superposition of the scattering intensities of the individual substructures that appear within the film, if the length scales of the distinct substructures are sufficiently separated. [4]

GISAXS experiments can also provide information in the vertical direction, such as correlated roughness. In general, resonant diffuse scattering from correlated interfaces appears in the vertical direction when a part of the roughness spectrum is transferred from one interface to another interface. Therefore, the distance between the interference fringes and the amplitude of the resonant diffuse scattering give information about the thickness of correlated layers and the spectrum, which is correlated.

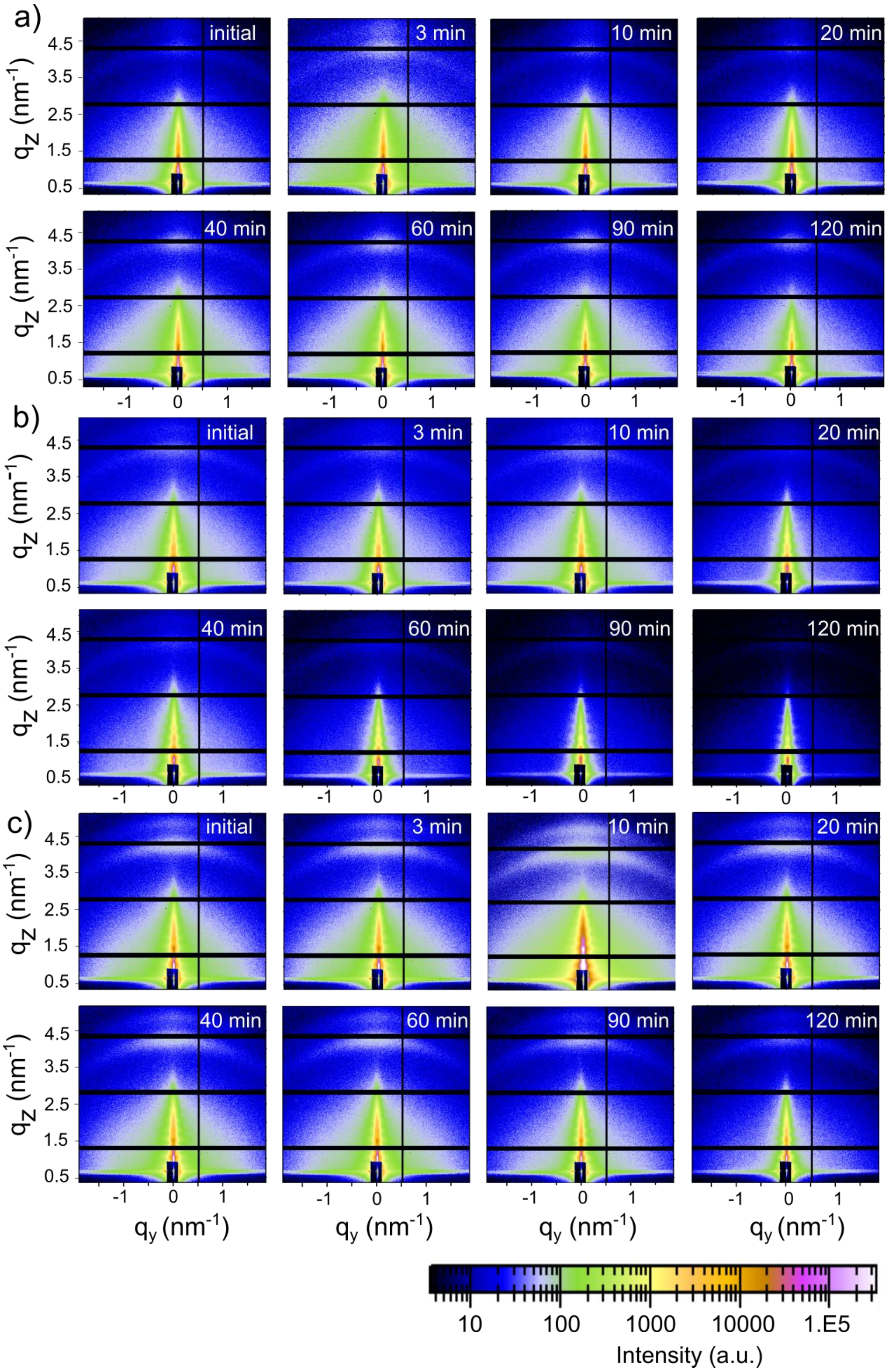

Figure S3. 2D GISAXS data of solar cells after different illumination times as indicated: a) device without additive, b) device with DIO additive, and c) device with CBA additive.

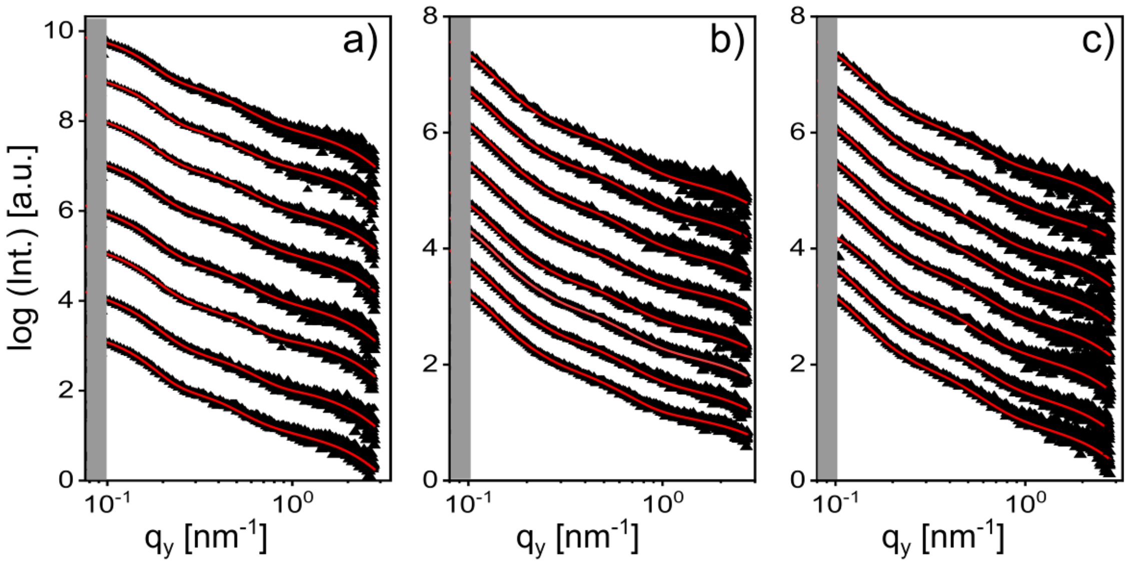

Figure S4. Horizontal line cuts of the 2D GISAXS data measured (symbols) after different operation times are shown together with fits (red lines): a) device without additive, b) device with DIO additive, and c) device with CBA additive. From bottom to top: GISAXS measurements after 0, 3, 10, 20, 40, 60, 90 and 120 minutes of device operation. The curves are shifted along the y axis for clarity. The q-range shielded by the beamstop is indicated by the gray area.

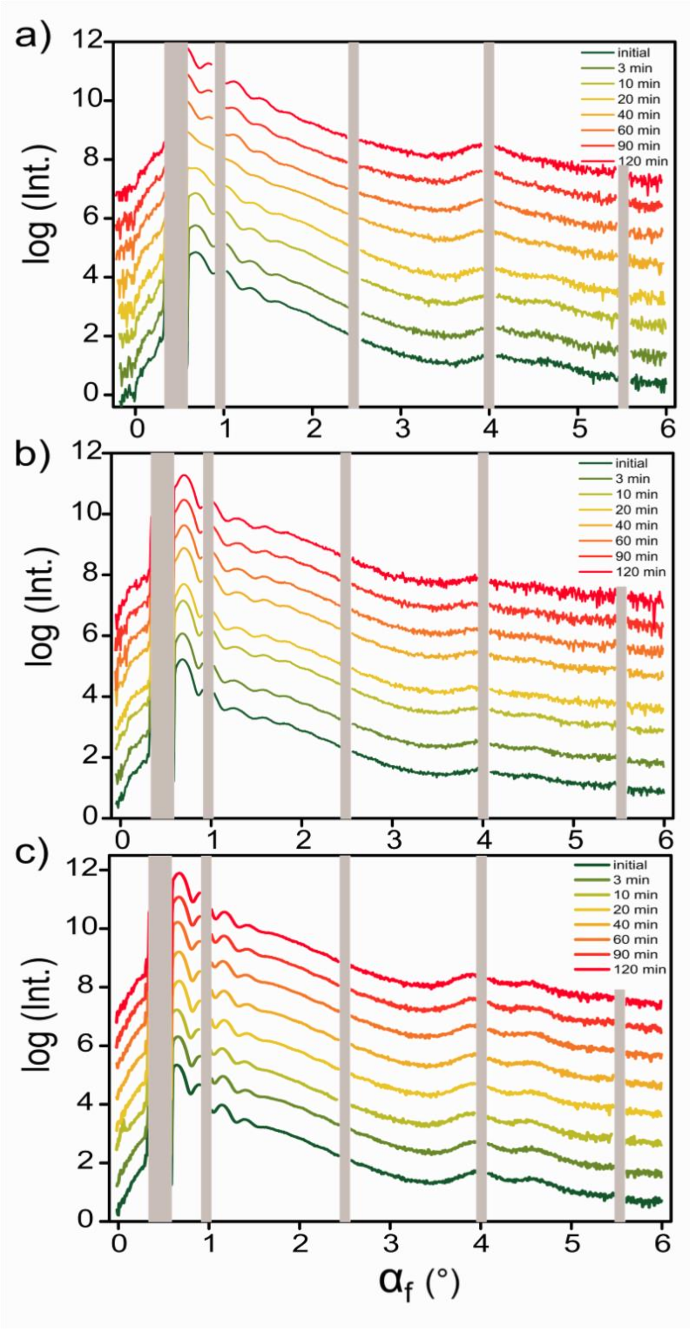

Figure S5. Vertical line cuts of the 2D GISAXS data measured after different operation times: a) device without additive, b) device with DIO additive, and c) device with CBA additive. The curves are shifted along the y axis for clarity. The non-accessible q-regions due to the beamstop shielding the detector and detector gaps are indicated by grayed-out areas.

GIWAXS analysis

Prior to the GIWAXS analysis, efficiency-, solid-angle- and χ-correction were performed on the raw GIWAXS data to obtain correct q_z vs. q_r images, because a distortion originating from the conflict between the spherical and flat hypersurfaces of the Ewald's sphere and the flat crystalline planes and q_x needs to be accounted for in GIWAXS measurements, especially for large exit angles.[5] In this work, the corrections are done by the aid of the GIXSGUI 1.6.1 software.[6] Subsequently, the reference GIWAXS data of the pure ITO substrate was subtracted from the 2D GIWAXS data to remove contributions from the substrate and background. Afterwards, cake cuts were extracted from the background-corrected data along $\boldsymbol{q}$ across the (100) PffBT4T-2OD Bragg peak in the perpendicular direction, as exampled in Figure S5a. At last, the cake cuts were fitted by Gaussian distributions to obtain the intensity, q-position and FWHM value of the (100) PffBT4T-2OD Bragg peak, as shown in Figure S5b. The crystalline grain sizes were evaluated via the Scherrer equation along the crystal direction [hkl] = [100]. The X-ray beam at the synchrotron source exhibits certain intensity fluctuation, which were accounted for by normalizing the overall scattering intensity for the intensity of the peak arising from the Kapton windows of the used setup (q = 0.5 Å).

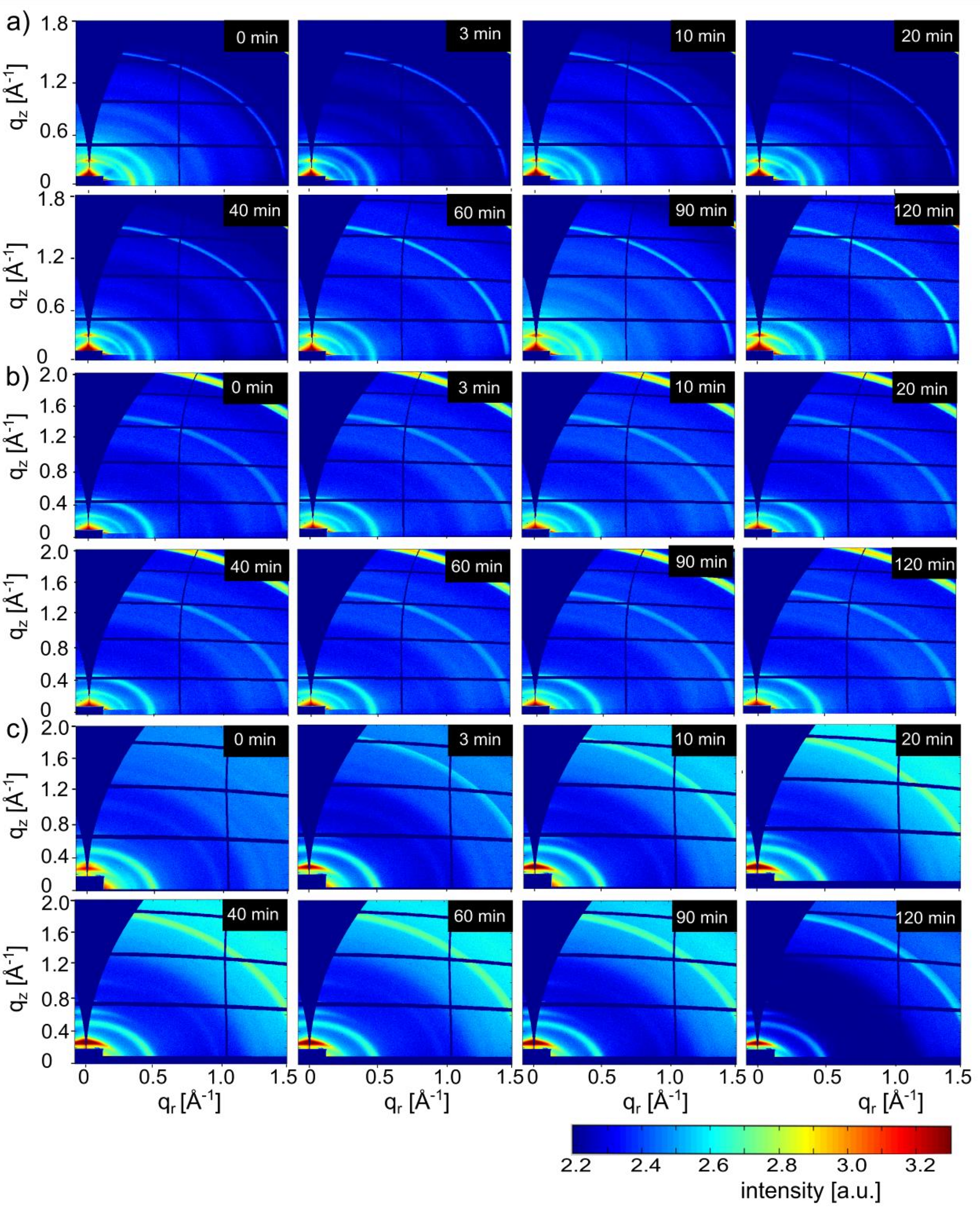

Figure S6. 2D GIWAXS data of solar cells after different illumination times as indicated: a) device without additive, b) device with DIO additive, and c) device with CBA additive.

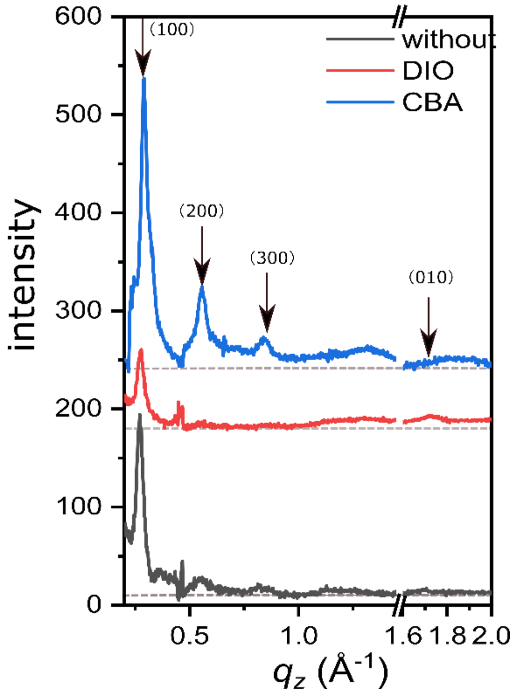

Figure S7. Vertical cake cuts of the initial GIWAXS data of all samples after correction: without additives (black line), with DIO (red line) and with CBA (blue line). The gray dashed lines are the baselines as a guide to the eye.

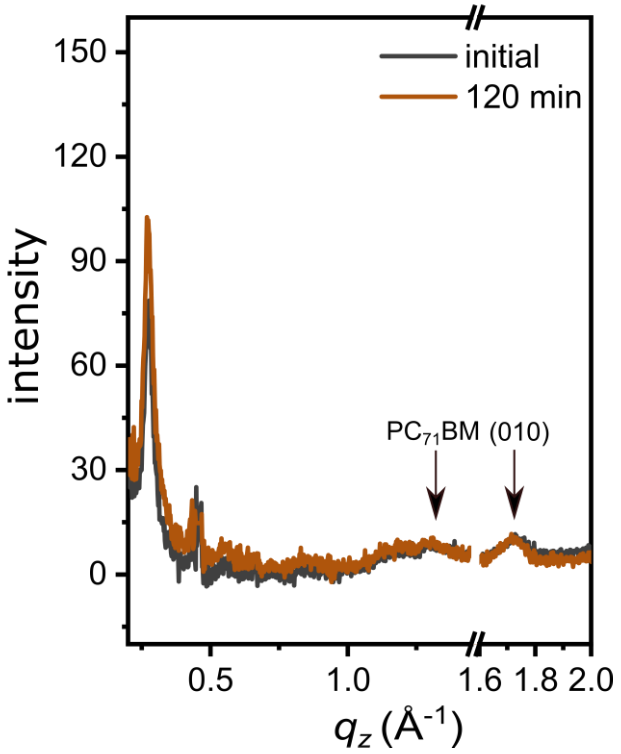

Figure S8. Vertical cake cuts of the initial (black) and the final (brown) GIWAXS data of the device with DIO additive after correction. The fullerene peak (q = 1.3 A^{-1}) and the polymer (010) Bragg peak are labelled.

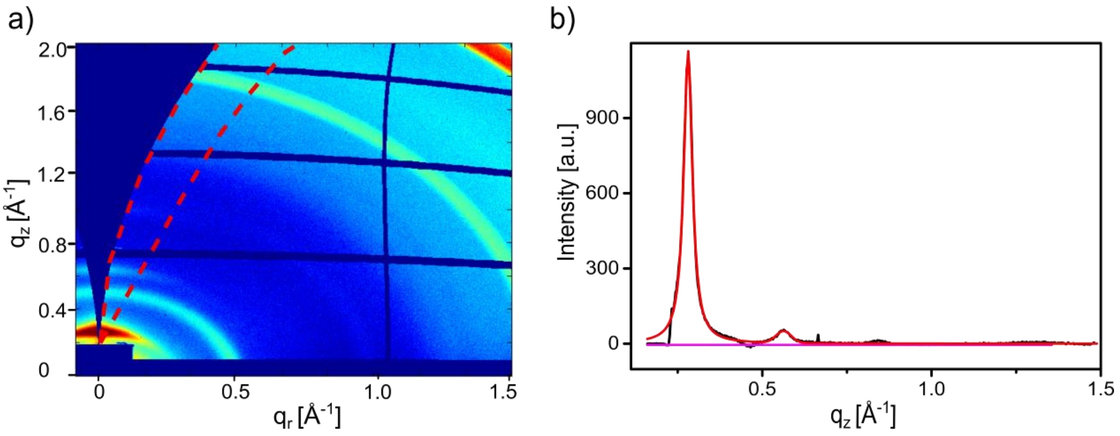

Figure S9. a) Exemplary 2D GIWAXS data set of the sample with CBA indicating the performed cake cut (red dash lines). b) Corresponding cake cut after background subtraction. The peaks are fitted by Gaussian functions. The red line stands for the fitting line, and the purple line is the baseline.

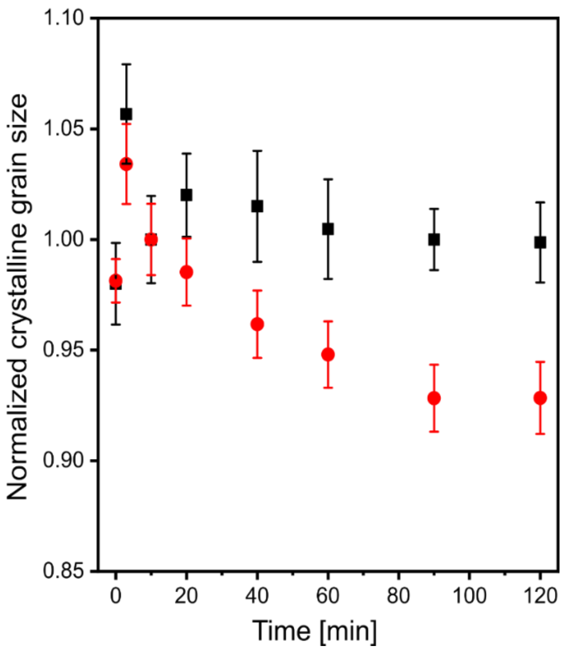

Figure S10. Time evolution of the polymer crystalline grain size of devices with DIO. Red data points correspond to the GIWAXS measurements under solar illumination, whereas black data points correspond to a control measurement in the dark.

Radiation damage analysis

Considering the potential radiation damage caused by the high-brilliance X-ray beam, a radiation damage test is performed on a device with DIO additive before the real in-operando measurements. 36 GISAXS measurements were continuously done at one fixed spot (around 5 mm away from the electrode) of a solar cell with 5 s exposure time for a single measurement without operating the solar cell. **Figure S11** shows the corresponding mapping of these measurements by showing the horizontal line cuts of the 2D GISAXS data of all measurements. No significant changes in the intensity during this exposure time are visible. A slight intensity difference is caused by the beam intensity fluctuation during the measurements.

During the in-operando measurements, the current-voltage measurements are performed periodically, but the X-ray scattering measurements are only carried out at selected times with a total dose of X-ray radiation below the limit determined from the radiation damage test. Moreover, we do not observe any additional changes on the temporal evolution of photovoltaic parameters caused by the presence of the X-ray beam. Thus, we conclude that the X-ray beam does not affect the device performance.

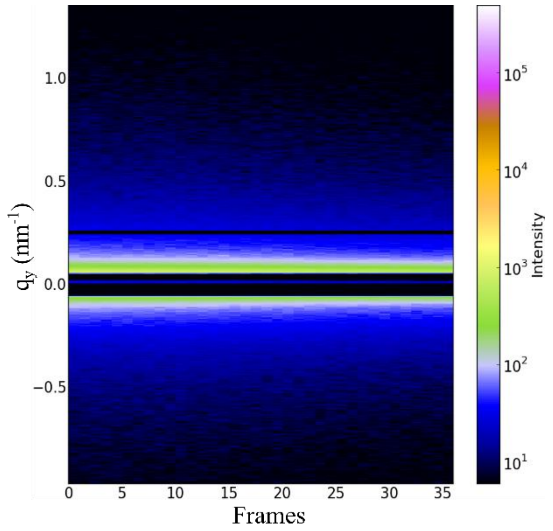

Figure S11. Color mapping of horizontal line cuts of the 2D GISAXS data of all radiation damage measurements on a device with DIO additive. The horizontal black stripes are due to the inter-module gaps of the detector and the beamstop.

References:

[1] D. Yang, F. C. Löhrer, V. Körstgens, A. Schreiber, S. Bernstorff, J. M. Buriak, P. Müller-Buschbaum, *ACS Energy Lett.* **2019**, *4*, 464.

[2] D. Gazzillo, A. Giacometti, R. G. Della Valle, E. Venuti, F. Carsughi, *J. Chem. Phys.* **1999**, *111*, 7636.

[3] G. L. Frontini, F. Otero, M. G. Messineo, G. E. Eliçabe, *Inverse Probl. Sci. Eng.* **2008**, *16*, 995.

[4] T. A. Ezquerra, M. C. Garcia-Gutierrez, A. Nogales, M. Gomez, *Applications of Synchrotron Light to Scattering and Diffraction in Materials and Life Sciences*, Springer Berlin Heidelberg, **2009**.

[5] J. L. Baker, L. H. Jimison, S. Mannsfeld, S. Volkman, S. Yin, V. Subramanian, A. Salleo, A. P. Alivisatos, M. F. Toney, *Langmuir* **2010**, *26*, 9146.

[6] Z. Jiang, *J. Appl. Crystallogr.* **2015**, *48*, 917.
